# LONG-TERM ASSOCIATION OF AN INTENSIVE LIFESTYLE INTERVENTION ON FRAILTY AND MULTIMORBIDITY

**DOI:** 10.1093/geroni/igad104.1700

**Published:** 2023-12-21

**Authors:** Felicia Simpson, Mark Espeland, Joni Evans, Karen Johnson, Judy Bahnson, Denise Houston, Mia Yang, Lynne Wagenknecht

**Affiliations:** Winston-Salem State University, Winston-Salem, North Carolina, United States; Wake Forest University School of Medicine, Winston-Salem, North Carolina, United States; Wake Forest School of Medicine, Winston-Salem, North Carolina, United States; University of Tennessee Health Science Center, Memphis, Tennessee, United States; Wake Forest School of Medicine, Winston-Salem, North Carolina, United States; Wake Forest University School of Medicine, Winston Salem, North Carolina, United States; Wake Forest School of Medicine, Winston-Salem, North Carolina, United States; Atrium Health/Wake Forest Baptist, Winston-Salem, North Carolina, United States

## Abstract

Multidomain lifestyle interventions may slow aging as captured by deficit accumulation frailty indices (FIs) and multimorbidity. We previously reported that the 10-year Look AHEAD multidomain intensive lifestyle intervention (ILI) that focused on caloric restriction, increased physical activity, improved diet, and cardiometabolic risk factor monitoring, significantly slowed the progression of both FI and a multimorbidity index during intervention delivery. We assessed whether these benefits endured after ILI delivery was terminated and the degree to which these benefits may be interrelated. The Look AHEAD Aging study enrolled N=1606 individuals, ages 63-94 years, who had been assigned a median of 20 years earlier (i.e., 10 years after ILI was terminated) to either the Look AHEAD ILI or its control condition of diabetes support and education (DSE). At the original Look AHEAD randomization, they had a mean (SD) 39-component FI score of 0.128 (0.056) and mean (SD) 9-component multimorbidity index of 1.63 (1.01), both balanced across intervention groups. When enrolled in the Look AHEAD Aging study, participants who had been assigned to ILI, compared with those assigned to DSE, averaged 6% lower FI scores (p<0.0001). They had also accrued 0.14 fewer additional age-related chronic diseases from baseline (p<0.0001). Relative benefits associated with ILI for these indices were similar for both genders and across the full cohort’s age range. Increases in FI and multimorbidity were highly correlated (r=0.55; p<0.001), similarly in both arms. Multidomain lifestyle interventions can result in persistent long-term benefits in two common markers of aging.

